# Assessment and Density Functional Theory of Bioactive Compounds of *Curcuma longa* L. Root Responsible for Its Cardio-Protective and Anti-Cancer Activities

**DOI:** 10.3390/ph19060834

**Published:** 2026-05-27

**Authors:** Ahmed Hemdan, Sylvester Nnaemeka Ugariogu, Bashayer D. Althufairi, Naser F. Al-Tannak

**Affiliations:** Department of Pharmaceutical Chemistry, College of Pharmacy, Health Science Center, Kuwait University, Jabriya, P.O. Box 24923, Safat 13110, Kuwait; ahmad.hemdan@ku.edu.kw (A.H.); mastersylvester@yahoo.com (S.N.U.); bashayer.althufairi@ku.edu.kw (B.D.A.)

**Keywords:** phytochemical, GC-MS, insilico studies, cardioprotective and anticancer properties, pharmacokinetic

## Abstract

**Background/Objectives:** Cardiovascular diseases (CVDs) and cancer remain major global health challenges and are among the leading causes of mortality worldwide, including in Kuwait. Medicinal plants are important sources of bioactive compounds with therapeutic potential. This study aimed to identify the phytochemical constituents of *Curcuma longa* L. root extract and evaluate their potential cardioprotective and anticancer activities using integrated computational approaches. **Methods:** Phytochemical profiling of *Curcuma longa* root extract was performed using gas chromatography–mass spectrometry (GC–MS). The identified compounds were evaluated through molecular docking against selected cardiovascular- and cancer-related targets, including HMG-CoA reductase, phosphoinositide 3-kinase (PI3K), cyclin-dependent kinase 6 (CDK6), and HER2 kinase receptors. Protein–ligand interactions were analyzed to determine binding stability. Biological activity prediction and pharmacokinetic properties were assessed using PASS prediction and SwissADME tools, while density functional theory (DFT) calculations were conducted to investigate electronic and quantum chemical characteristics associated with ligand reactivity. **Results:** GC–MS analysis identified seventeen phytochemical constituents with retention times ranging from 7.57 to 32.70 min. The major compounds detected were 2-oxo-cyclooctaneacetic acid (30.88%), curlone (20.99%), and tumerone (13.85%). Molecular docking revealed favorable binding affinities for α-curcumene, caryophyllene, bergamotene, cyclohexene derivatives, tumerone, curlone, and (6R,7R)-bisabolone against the selected targets, with interaction profiles comparable to reference drugs. PASS and SwissADME analyses indicated promising biological activities, acceptable drug-likeness, and favorable pharmacokinetic properties. DFT analysis demonstrated that curlone and tumerone possessed stable electronic configurations and favorable reactivity profiles. **Conclusions:** The findings suggest that bioactive compounds from *Curcuma longa* may serve as promising lead candidates for the development of cardioprotective and anticancer agents. However, further experimental validation through in vitro and in vivo studies is required to confirm these computational predictions.

## 1. Introduction

Cardiovascular diseases (CVDs) and cancer remain the leading causes of global mortality, accounting for a significant proportion of deaths worldwide [1]. The World Health Organization reports that CVDs are responsible for approximately 17.9 million deaths annually, with myocardial infarction and stroke contributing to nearly 80% of cases [2]. In Kuwait, cardiovascular disease (40%) and cancer (22%) represent the major causes of mortality, highlighting a significant regional health burden [1]. These conditions are strongly associated with modifiable risk factors such as unhealthy diet, physical inactivity, tobacco use, alcohol consumption, and environmental pollution, which contribute to hypertension, obesity, dyslipidemia, and diabetes [2,3].

Cancer is a complex genetic disorder characterized by uncontrolled cell proliferation and dysregulated molecular signaling pathways. Unlike many diseases, cancer involves multiple biological targets, making therapeutic intervention highly challenging [4]. Therefore, multi-target drug discovery approaches are essential for identifying compounds capable of modulating key disease pathways.

Natural products remain a cornerstone of drug discovery due to their structural diversity and wide pharmacological potential [5]. Among these, *Curcuma longa* L. (turmeric), a medicinal plant from the *Zingiberaceae* family, has been extensively used in traditional medicine systems such as Ayurveda and Unani for the treatment of inflammation, liver disorders, infections, wounds, and chronic diseases [6,7]. It is also widely used as a dietary spice and functional food across Asia [7].

The rhizome of *Curcuma longa* contains diverse bioactive phytochemicals, including curcuminoids, terpenoids, phenolics, and essential oils, which contribute to its wide pharmacological effects [7,8]. Numerous studies have demonstrated its antioxidant, anti-inflammatory, antimicrobial, anticancer, anti-obesity, neuroprotective, and cardioprotective activities [6,7,8]. These properties make turmeric a promising candidate for further pharmacological investigation.

Curcumin, the principal bioactive compound of turmeric, has been widely studied for its therapeutic effects. It modulates multiple molecular pathways involved in inflammation, oxidative stress, apoptosis, and immune regulation [9]. Phenolic acids derived from natural sources also demonstrate cardioprotective, anti-inflammatory, and anticancer activities by regulating apoptosis, angiogenesis, and cytokine signaling pathways [10]. Furthermore, *Curcuma longa* extracts exhibit broad pharmacological effects, including cardioprotective, hepatoprotective, antimicrobial, and neuroprotective activities [11].

Recent evidence has strengthened the clinical relevance of curcumin in cardiovascular disease management. Li et al. (2023) reported that curcumin significantly improves myocardial ischemia/reperfusion injury through antioxidant and anti-inflammatory mechanisms [12]. Similarly, Wang et al. (2024) demonstrated its protective role in diabetic cardiomyopathy by regulating oxidative stress, apoptosis, and inflammatory pathways. These findings highlight curcumin’s potential in cardiometabolic disease prevention [13].

In oncology, turmeric-derived compounds have demonstrated significant therapeutic potential. Cozmin et al. (2024) reported anticancer, radioprotective, and anti-inflammatory properties of turmeric compounds [14], while Amaroli et al. (2024) emphasized curcumin’s role in cancer management through modulation of apoptosis and tumor signaling pathways [15]. Gutsche et al. (2025) further supported its use as a complementary agent in oncological therapy [16]. Additionally, Kaur et al. (2024) highlighted curcumin analogs with enhanced antioxidant and anticancer properties [17], and Jafari et al. (2024), based on 103 randomized controlled trials, confirmed its safety and therapeutic efficacy in human health [18].

Hypercholesterolemia is a major risk factor for cardiovascular disease and is closely associated with atherosclerosis, myocardial infarction, and stroke [19,20]. The enzyme 3-hydroxy-3-methylglutaryl coenzyme A (HMG-CoA) reductase plays a central role in cholesterol biosynthesis and is a primary therapeutic target for lipid-lowering agents [20]. Although statins are effective inhibitors, their long-term use is associated with adverse effects, necessitating safer plant-derived alternatives [20].

In cancer biology, key signaling pathways such as phosphoinositide 3-kinase (PI3K)/Akt/mTOR regulate cell survival, proliferation, and metabolism and are important therapeutic targets [21,22]. Cyclin-dependent kinases (CDK4/6) regulate cell cycle progression and are implicated in cancer development and metastasis [23,24]. Additionally, epidermal growth factor receptor (EGFR) and human epidermal growth factor receptor 2 (HER2) are overexpressed in several cancers and represent validated targets for anticancer drug development [25].

Recent computational approaches in drug discovery, including molecular docking, PASS prediction, and ADMET profiling, have become essential tools for screening bioactive compounds and identifying potential drug candidates efficiently [26,27,28,29]. These methods significantly reduce the time, cost, and limitations associated with experimental drug development.

Given the rich phytochemical profile and reported pharmacological activities of *Curcuma longa*, it represents a promising source of multi-target bioactive compounds for both cardiovascular and cancer therapy. Therefore, this study aims to identify phytochemicals from turmeric using GC-MS analysis and evaluate their cardioprotective and anticancer potential through in silico molecular docking, pharmacokinetic (ADMET) prediction, and PASS analysis. DFT studies further provide detailed information on the electronic properties, stability, and chemical reactivity of bioactive molecules. This integrated computational approach provides mechanistic insight into compound–target interactions and supports the identification of potential lead compounds for future drug development.

## 2. Result

### 2.1. Result of GCMS Profiling

**Figure 1:**
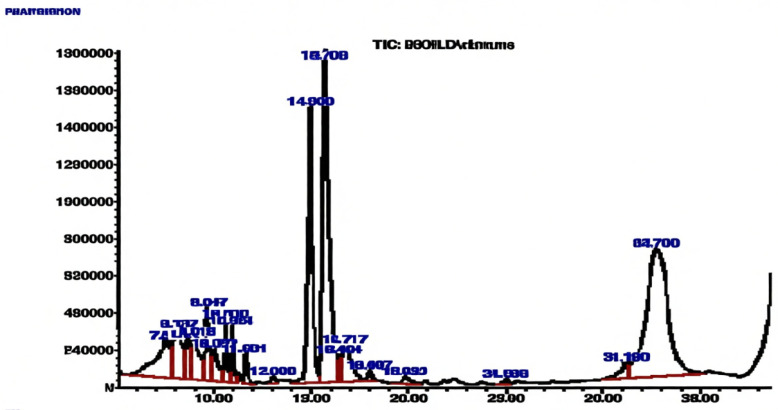
The GC-MS spectra of ethanolic turmeric extract.

The result of the GC-MS analysis for the phytochemical composition of turmeric is shown below in Figure 1 and Tables S1 and S2 in the Supplementary Documents.

GC–MS analysis of the ethanolic extract of *Curcuma longa* rhizome revealed a total of 17 bioactive phytochemical constituents with retention times ranging from 7.57 to 32.7 min.

The chromatographic profile showed that the extract is rich in terpenoids and oxygenated sesquiterpenes. The major identified compounds included: 2-oxo-cyclooctaneacetic acid (30.88%), Curlone (20.99%), Turmerone (13.85%). Other minor constituents such as curcumene, caryophyllene, bergamotene, and bisabolone derivatives. The high abundance of oxygenated sesquiterpenes suggests strong bioactivity potential, consistent with previously reported phytochemical profiles of *Curcuma longa* [7,11,30].

### 2.2. Result of Molecular Docking

Molecular docking was performed to evaluate binding interactions between identified phytochemicals and selected cardiovascular and cancer-related protein targets: HMG-CoA reductase (IT02), PI3Kγ (4URK), CDK6 (4EZ5), and HER2 kinase (3RCD).

The results of molecular docking for cardiovascular and cancer diseases are reported below in Table 1 and Table 2, Tables S3 and S4 in Supplementary Materials.

**Figure 2:**
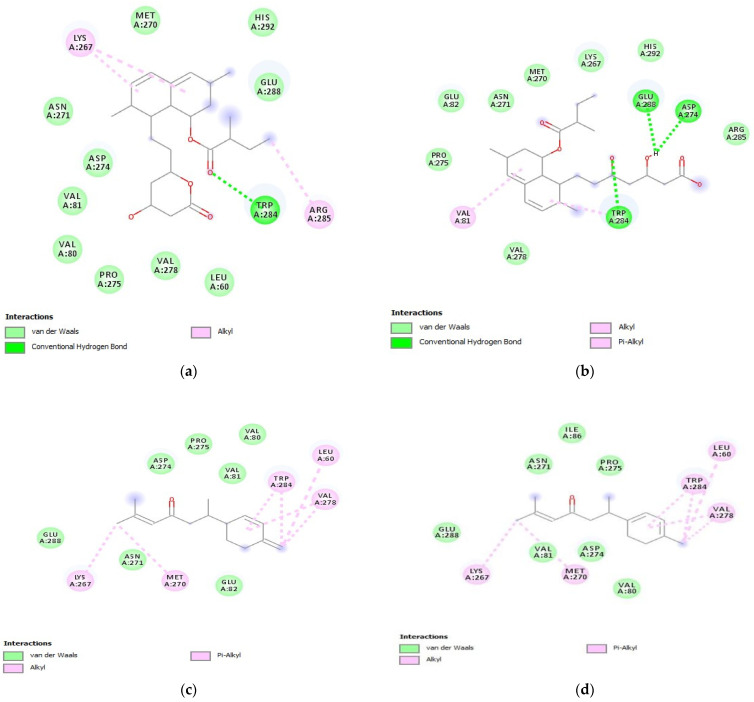

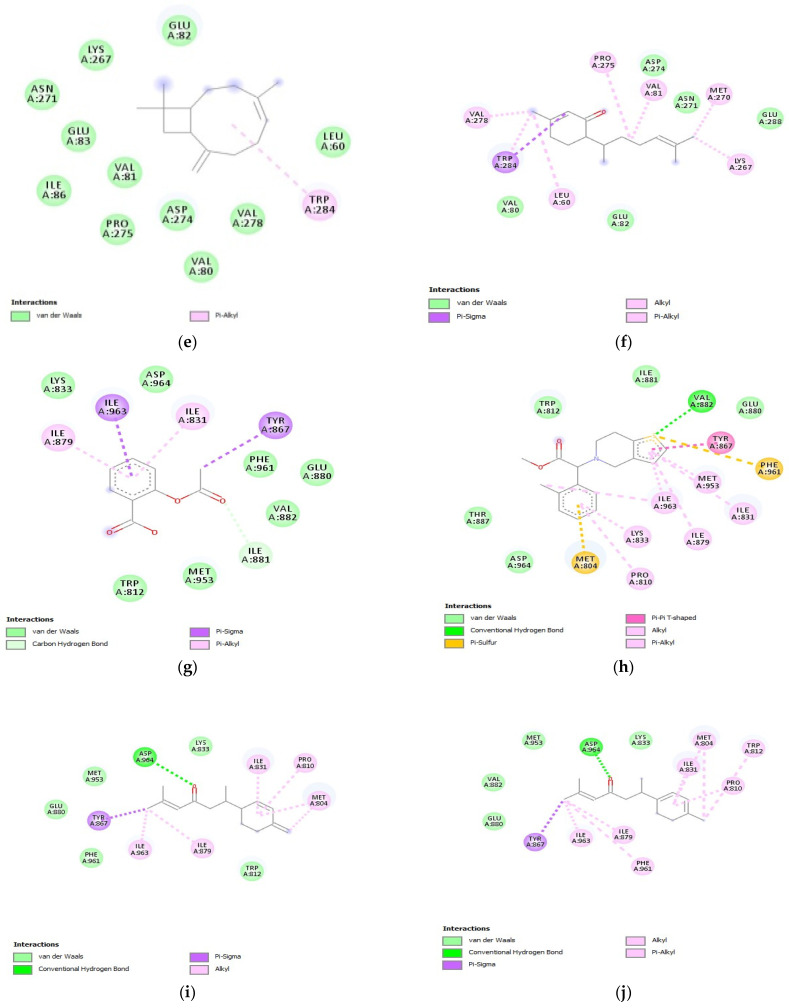

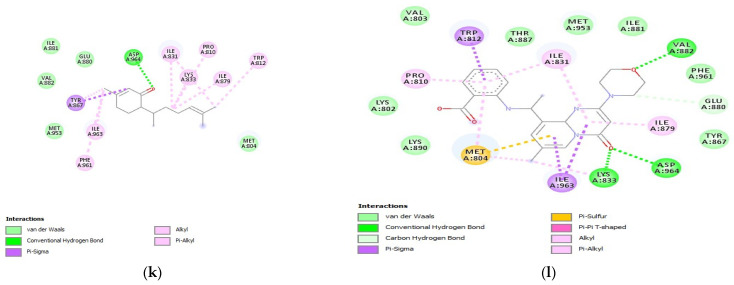
(**a**) Interaction of 1T02 with Lovastatin, (**b**) Interaction of 1T02 with Cocrystal ligand of 1T02, (**c**) Interaction of 1T02 with Curlone, (**d**) Interaction of 1T02 with Tumerone, (**e**) Interaction of 1T02 with Caryophyllene, (**f**) Interaction of 1T02 with (6R,7R)-Bisabolone, (**g**) Interaction of 4URK with Aspirin, (**h**) Interaction of 4URK with Clopidogrel, (**i**) Interaction of 4URK with Curlone, (**j**) Interaction of 4URK with Tumerone, (**k**) Interaction of 4URK with Bisabolone, (**l**) Interaction of 4URK with Cocrystal ligand of 4URK.

The Figure 2a–l below show the interactions of the cardiovascular disease related protein 1T02 and 4URK with standard drugs, cocrystaline ligand, and the compounds with the best binding affinities.

**Figure 3:**
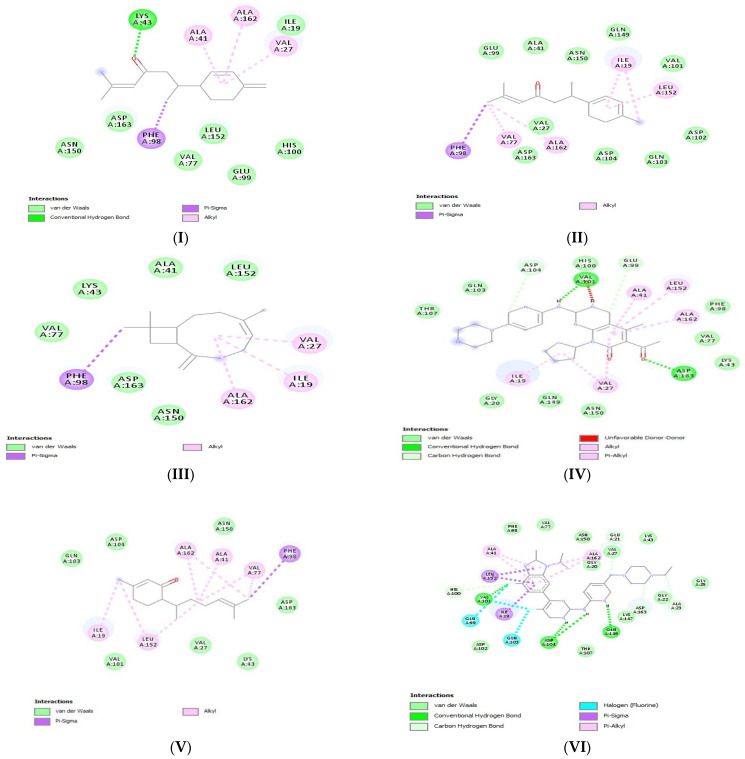

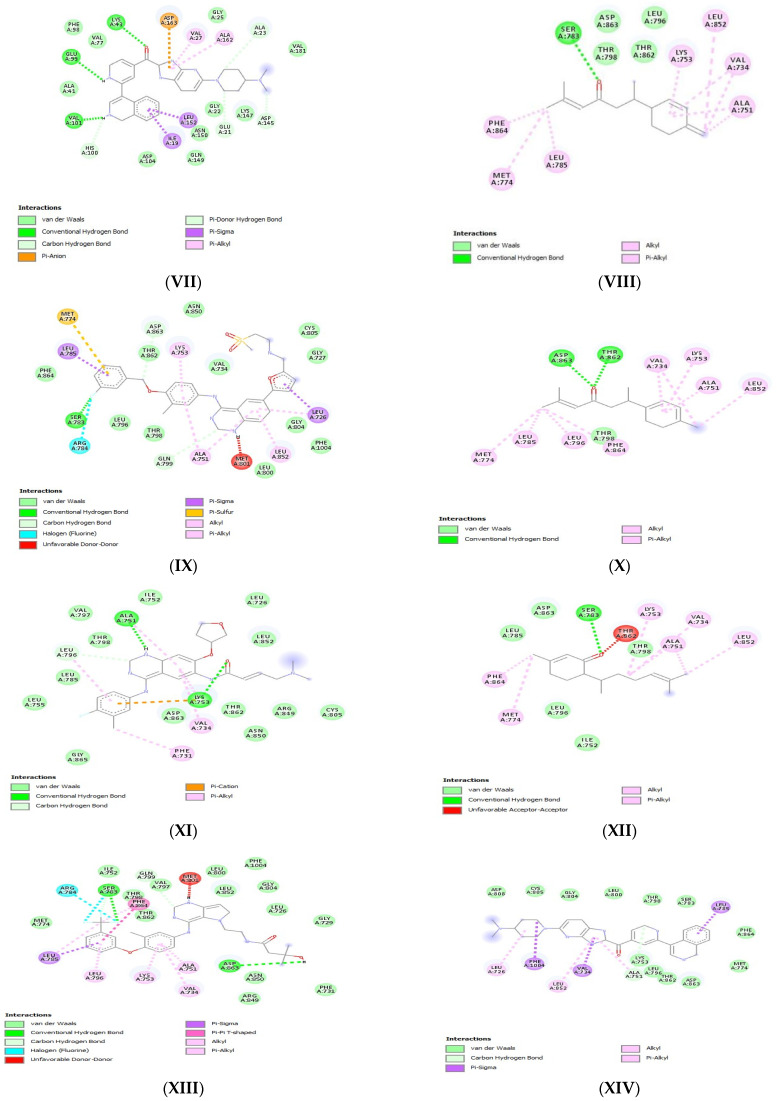
(**I**) Interaction of 4EZ5 with Curlone, (**II**) Interaction of 4EZ5 with Tumerone, (**III**) Interaction of 4EZ5 with Caryophyllene, (**IV**) Interaction of 4EZ5 with drug palbociclib, (**V**) Interaction of 4EZ5 with Bisabolene, (**VI**) Interaction of 4EZ5 with drug Abemaciclib, (**VII**) Interaction of 4EZ5 with Cocrystalline ligand of 4EZ5, (**VIII**) Interaction of 3RCD with Curlone, (**IX**) Interaction of 3RCD with lapatinib, (**X**) Interaction of 3RCD with Tumerone, (**XI**) Interaction of 3RCD with drug Afatinib, (**XII**) Interaction of 3RCD with Bisabolone, (**XIII**) Interaction of 3RCD with TAK-285 (3RCD), (**XIV**) Interaction of 3RCD with Cocrystalline ligand of 4EZ5.

The Figure 3I–XIV below show the interactions of the cancer related proteins 4EZ5 and 3RCD with standard drugs, cocrystaline ligands, and the compounds with the best binding affinities.

#### 2.2.1. Binding Affinity Profile

All screened compounds exhibited variable binding affinities across the selected targets. The most significant interactions were observed with: Curlone, Turmerone, Curcumene, Caryophyllene, Bergamotene, Bisabolone derivatives. These compounds demonstrated binding energies comparable to reference inhibitors co-crystallized in the respective protein structures.

Notably, Curlone and Turmerone consistently showed the highest docking stability across multiple targets, indicating multi-target potential. Standard drug comparators (statins, AZD6482, lapatinib-like scaffolds, and CDK inhibitors) showed expected high binding affinities, validating docking reliability.

#### 2.2.2. Interaction Analysis

Docking visualization revealed that active compounds formed: Hydrogen bonds with key catalytic residues. Hydrophobic interactions within binding pockets, π–alkyl, and van der Waals interactions stabilize ligand orientation. In particular, Curlone and Turmerone exhibited stable binding conformations within the HMG-CoA reductase active site (cholesterol biosynthesis pathway), PI3Kγ catalytic domain (cardiovascular signaling pathway), CDK6 ATP-binding pocket (cell cycle regulation pathway), and HER2 kinase domain (oncogenic signaling pathway). These findings suggest potential dual cardioprotective and anticancer activity mediated through multi-target binding.

### 2.3. PASS-Based Biological Activity Prediction

PASS analysis was used to estimate the probability of selected biological activities based on structure–activity relationships as reported in Tables S5–S12 in Supplementary Materials.

The selected compounds demonstrated predicted activities associated with anti-inflammatory, antineoplastic (anticancer) potential, cardioprotective activity, antioxidant effects, kinase-inhibition-related activity, apoptotic-agonist activity, cholesterol-antagonist activity, MMP9 suppression, and fibrinolytic activity. PASS analysis of top-ranked compounds indicated a high probability of pharmacological activity (Pa > Pi) for several biological effects. Key predicted activities included: Among all compounds, Turmerone and Curlone showed the highest predicted activity spectrum, supporting their strong docking performance.

### 2.4. ADMET and Drug-likeness Evaluation

The selected compounds were further evaluated for their pharmacokinetic behavior and drug-likeness properties using SwissADME predictions (Table 3 and Table 4).

The investigated phytochemicals generally exhibited molecular weights below 250 g/mol, moderate lipophilicity, and acceptable topological polar surface area values, supporting their potential suitability for oral drug development. The SwissADME analysis revealed that most bioactive compounds satisfied Lipinski’s Rule of Five, indicating acceptable oral drug-likeness. Key pharmacokinetic observations included: Moderate to high gastrointestinal absorption for major compounds, Favorable lipophilicity profiles for membrane permeability, No significant violation of drug-likeness rules for lead compounds, Low predicted toxicity risk for top candidates. Among the screened molecules, Curlone and Turmerone demonstrated the most balanced ADMET profiles, supporting their potential as drug leads.

### 2.5. Density Functional Theory (DFT) Calculations

**Figure 4:**
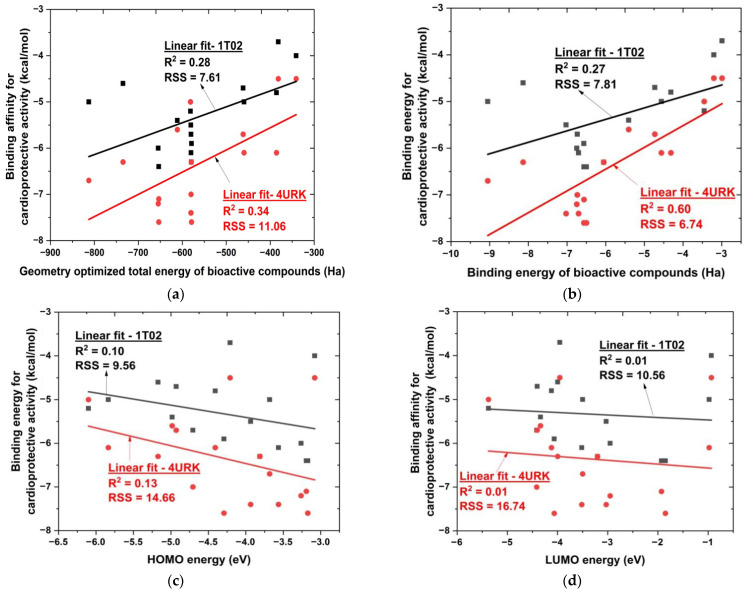
The relationship between binding affinity/docking scores of the cardioprotective enzymatic activity with (**a**) optimized energies values, (**b**) binding energies, (**c**) HOMO energy, (**d**) LUMO of the compounds.

**Figure 5:**
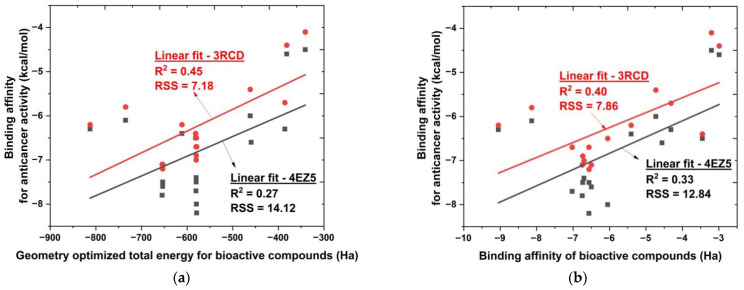

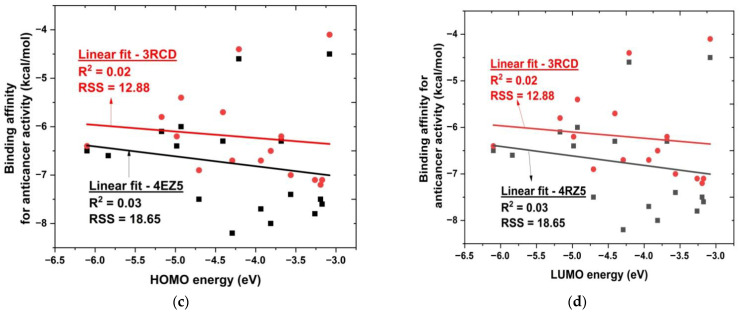
The relationship between binding affinity/docking scores of the anticancer enzymatic activity with (**a**) optimized energy values, (**b**) binding energies, (**c**) HOMO energy, (**d**) LUMO of the compounds.

DFT calculations were performed to investigate the electronic stability and reactivity descriptors of the selected phytochemicals (Table 5). Parameters including total energy of bioactive compound (Figure 4a and Figure 5a), Binding energy of bioactive compounds (Figure 4b and Figure 5b), HOMO (Figure 4c and Figure 5c), LUMO (Figure 4d and Figure 5d), energy gap, ionization energy, electron affinity, global hardness, and softness were analyzed to explore possible relationships with binding affinity.

#### 2.5.1. Frontier Molecular Orbital Analysis

HOMO–LUMO energy gap analysis indicated that Curlone and Turmerone possess moderate chemical reactivity and stable electronic configurations. Lower energy gaps suggest higher reactivity, which may enhance protein binding potential.

#### 2.5.2. Global Reactivity Descriptors

Calculated descriptors showed: Moderate ionization potential (IE) across compounds, Stable electron affinity (EA) values. Balanced hardness (η) and softness (S), indicating structural stability with sufficient reactivity for biological interaction. These quantum chemical properties support docking results and indicate suitability for biological activity.

### 2.6. Statistical and Multivariate Analysis

Principal Component Analysis (PCA) was performed to evaluate relationships between: Binding affinity scores, Molecular descriptors, and Quantum chemical parameters.

#### 2.6.1. PCA Results

**Figure 6:**
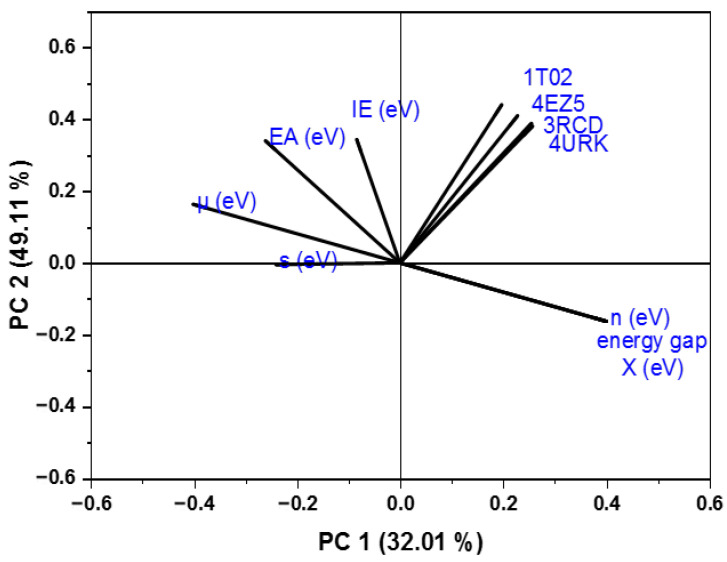
PCA of binding affinity/docking scores and remaining quantum chemical parameters.

Compounds clustered into distinct groups based on structural similarity and binding behavior as shown in Table 5 and Figure 6. Curlone and Turmerone were positioned in the high-contribution quadrant, indicating strong correlation with binding affinity and pharmacological activity.

#### 2.6.2. Regression Analysis

Linear regression demonstrated a positive correlation between: Docking binding affinity and HOMO energy values, Binding affinity and molecular stability descriptors. This suggests that electronic properties significantly influence ligand–protein interaction strength.

### 2.7. Integrated Multi-Target Pharmacological Profile

Overall integration of GC–MS, molecular docking, PASS, ADMET, and DFT results indicates that *Curcuma longa* contains multiple bioactive compounds capable of interacting with both cardiovascular and cancer-related targets. Curlone and Turmerone emerged as the most promising dual-activity candidates. Multi-target modulation suggests potential synergistic therapeutic effects.

These findings support the hypothesis that *Curcuma longa* phytochemicals may serve as lead scaffolds for developing novel cardioprotective and anticancer agents.

## 3. Discussion

The present study integrates GC–MS profiling, molecular docking, PASS prediction, ADMET evaluation, density functional theory (DFT), and multivariate statistics to investigate the cardioprotective and anticancer potential of *Curcuma longa* rhizome phytochemicals. The multi-layered computational approach provides a mechanistic understanding of how turmeric-derived compounds may interact with key molecular targets involved in cardiovascular diseases (CVDs) and cancer progression.

### 3.1. Phytochemical Profile and Biological Relevance

GC–MS analysis revealed a phytochemical profile dominated by oxygenated sesquiterpenes, particularly curlone, turmerone, curcumene, and related derivatives. These findings are consistent with previous reports describing *Curcuma longa* as a rich source of bioactive terpenoids with broad pharmacological properties [7,11,30].

Terpenoid-rich extracts have been widely associated with anti-inflammatory, antioxidant, and anticancer activities, largely due to their ability to modulate oxidative stress and inflammatory signaling pathways [8,31]. The high abundance of curlone and turmerone in this study supports their potential contribution to the observed multi-target pharmacological activity.

### 3.2. Multi-Target Molecular Docking and Mechanistic Insights

Molecular docking results demonstrated that several phytochemicals, particularly curlone and turmerone, exhibited strong binding affinities across multiple disease-relevant targets, including HMG-CoA reductase, PI3Kγ, CDK6, and HER2 kinase. This multi-target binding behavior is highly relevant in complex diseases such as CVDs and cancer, which are regulated by interconnected molecular signaling networks rather than single targets.

**Cardiovascular relevance**

Binding to HMG-CoA reductase suggests a potential role in cholesterol biosynthesis inhibition, a validated therapeutic strategy in hypercholesterolemia and atherosclerosis management [19,20]. Similarly, interactions with PI3Kγ indicate possible modulation of platelet activation and vascular inflammation, processes central to cardiovascular pathology [21,32].

**Anticancer relevance**

Strong binding to CDK6 and HER2 indicates that turmeric phytochemicals may interfere with cell cycle progression (CDK4/6 axis) and oncogenic receptor tyrosine kinase signaling (HER2 pathway). These pathways are critical in tumor proliferation and survival, particularly in breast and solid tumors [23,25]. The observed multi-target inhibition is consistent with emerging strategies in cancer drug discovery that prioritize network pharmacology over single-target inhibition.

### 3.3. PASS Prediction Supports Broad Pharmacological Potential

In PASS analysis, the probability of activity (Pa) and probability of inactivity (Pi) values provide probabilistic estimates rather than experimental confirmation of biological function. Compounds with Pa values greater than 0.70 are generally considered more likely to demonstrate biological activity experimentally, particularly when Pa substantially exceeds Pi values [33]. In the present study, the selected compounds exhibited Pa values greater than 0.70 for several predicted cardioprotective and anticancer activities, while corresponding Pi values remained comparatively low, supporting moderate predictive confidence.

Nevertheless, PASS predictions should be interpreted cautiously because they are derived from computational training datasets and cannot substitute for biochemical or cellular validation. The PASS results, therefore, serve only as supportive evidence complementing the docking and ADMET analyses rather than definitive proof of therapeutic efficacy.

Among the compounds, tumerone, curlone, bisabolone, and caryophyllene demonstrated comparatively broader predicted activity spectra, involving apoptosis induction, anti-inflammatory effects, and MMP9 suppression, which are mechanistically relevant to both cancer progression and cardiovascular pathology. These multitarget predictions align with previous reports describing the pleiotropic biological effects of turmeric-derived sesquiterpenoids [31,34].

PASS analysis further supported docking outcomes by predicting high probabilities for anti-inflammatory, anticancer, antioxidant, and cardioprotective activities among major compounds. The agreement between PASS prediction and docking results strengthens the reliability of the identified leads. Such computational convergence has been previously reported for natural products, where structural similarity to known bioactive molecules often translates into broad pharmacological potential [33].

### 3.4. ADMET Profile Suggests Drug-like Behavior

Most compounds satisfied Lipinski, Ghose, Veber, and Egan criteria, although all compounds failed the Muegge filter. This finding suggests that while the compounds possess several favorable drug-like characteristics, they may still require structural optimization to fully satisfy broader medicinal chemistry criteria. Therefore, the compounds should not be described as fully drug-like without qualification. Low gastrointestinal absorption should not automatically be considered advantageous. High GI absorption is generally desirable for orally administered drugs because it supports systemic bioavailability. In this study, tumerone, curlone, and bisabolone exhibited relatively favorable GI absorption profiles compared with other identified compounds. Likewise, the absence of BBB permeability may be beneficial for compounds intended for peripheral cardiovascular applications because it may reduce CNS-associated adverse effects; however, BBB permeability could be advantageous for therapies targeting brain tumors or neurological diseases. Therefore, BBB penetration should be interpreted according to the therapeutic context rather than as an inherently favorable or unfavorable property [26].

The investigated compounds also demonstrated relatively low CYP450 inhibitory tendencies, particularly toward CYP1A2 and CYP3A4, suggesting a potentially lower risk of drug–drug interactions. Nevertheless, several compounds showed predicted inhibition of CYP2C9 and CYP2C19 isoforms, indicating that metabolic interactions may still occur and should be experimentally evaluated in future pharmacological studies. Importantly, all selected compounds showed zero PAINS alerts, suggesting a relatively low probability of nonspecific assay interference. The synthetic accessibility values ranging from 2.02 to 4.68 also indicate that the compounds may be chemically accessible for future structural optimization studies. ADMET evaluation indicated that most active compounds, particularly curlone, turmerone, and bisabolone, exhibit favorable pharmacokinetic properties, including acceptable gastrointestinal absorption, lipophilicity, moderate medicinal chemistry properties, and compliance with Lipinski’s Rule of Five. These results are important because many promising phytochemicals fail during drug development due to poor pharmacokinetic behavior. The observed drug-likeness suggests that these compounds could serve as viable scaffolds for further optimization [26,27,28].

However, it is important to note that computational ADMET predictions represent early-stage screening and must be validated experimentally.

### 3.5. Quantum Chemical Properties and Reactivity Insights

The total energy values indicated that the geometry-optimized structures were energetically stable. However, regression analyses revealed weak correlations between total energy and docking scores (R^2^ < 0.45), suggesting that total energy alone is not a reliable predictor of ligand binding efficiency. Similar weak-to-moderate correlations were observed for HOMO, LUMO, and the energy gap values.

The frontier molecular orbital analysis showed that compounds with smaller HOMO–LUMO gaps generally possessed greater theoretical chemical reactivity. Cyclohexene derivative exhibited the smallest energy gap (0.05 eV), suggesting enhanced electronic flexibility. However, because the observed correlations between frontier orbital parameters and docking scores were weak, these descriptors should not be interpreted as direct predictors of biological activity. Instead, they provide supportive information regarding molecular reactivity and electron transfer tendencies.

Likewise, global softness and hardness values were evaluated based on HSAB theory. Softer molecules theoretically exhibit greater electron-donating capacity and stronger intermolecular interactions [35,36,37]. Although some compounds with higher softness values demonstrated moderate docking affinity, the correlations remained insufficiently strong to support definitive structure–activity conclusions.

Principal component analysis revealed partial clustering between electronic descriptors and binding affinity values, indicating that electronic properties may collectively contribute to ligand–protein interactions. However, steric effects, conformational flexibility, hydrophobic interactions, and receptor topology likely play equally important roles in determining docking behavior.

Overall, the DFT calculation provided additional insight into the electronic behavior of the phytochemicals and supports the view that electronic descriptors may assist in understanding ligand reactivity and interaction potential, but should not be considered standalone predictors of therapeutic activity in the absence of experimental validation. Moderate HOMO–LUMO energy gaps observed in curlone and turmerone suggest a balance between chemical stability and reactivity, which is favorable for ligand–protein interactions.

Global reactivity descriptors (hardness and softness) further indicated that these compounds possess sufficient electronic flexibility to interact with diverse protein targets. This supports their observed multi-target binding behavior in docking simulations.

Quantum chemical descriptors have been widely used to explain structure–activity relationships in natural product-based drug discovery and complement docking-based predictions [38,39].

### 3.6. Integrated Multi-Omics Computational Strategy

One of the key strengths of this study is the integration of multiple computational approaches:

GC–MS for compound identification, Molecular docking for target interaction analysis, PASS for biological activity prediction, ADMET for pharmacokinetic screening, DFT for electronic structure validation. This integrated pipeline provides a more reliable prediction framework compared to single-method studies and aligns with current trends in in silico drug discovery.

### 3.7. Pharmacological Implications and Dual Therapeutic Potential

The combined results suggest that *Curcuma longa* phytochemicals may exert dual cardioprotective and anticancer effects through: inhibition of cholesterol biosynthesis (HMG-CoA reductase), modulation of inflammatory and thrombosis pathways (PI3K signaling), suppression of cell cycle progression (CDK6), and inhibition of oncogenic receptor signaling (HER2). This dual-target activity is particularly important given the increasing evidence linking chronic inflammation, dyslipidemia, and cancer progression as interconnected biological processes.

### 3.8. Study Limitations

Despite promising findings, this study is limited by its purely computational nature. Molecular docking and in silico predictions do not fully capture biological complexity, including metabolism, bioavailability in vivo, and off-target effects. Additionally, GC–MS profiling focuses primarily on volatile and semi-volatile compounds; non-volatile bioactive constituents may not have been fully captured.

Therefore, experimental validation using in vitro enzymatic assays and in vivo disease models is necessary to confirm the predicted pharmacological effects.

### 3.9. Future Perspectives

Future studies should focus on: in vitro validation of HMG-CoA reductase, PI3K, CDK6, and HER2 inhibition, molecular dynamics simulations for stability confirmation, structural optimization of curlone and turmerone derivatives formulation studies to improve bioavailability, and clinical relevance assessment in disease models. Such approaches will help translate computational predictions into clinically relevant therapeutic candidates.

**Overall Significance**

This study demonstrates that *Curcuma longa* contains multiple bioactive compounds with strong potential for multi-target cardioprotective and anticancer therapy, with curlone and turmerone emerging as the most promising lead candidates.

## 4. Materials and Methods

### 4.1. Plant Material Collection and Sample Preparation

Fresh rhizomes of *Curcuma longa* L. were purchased from Nesto supermarket, Kuwait City, Kuwait. The plant material was washed thoroughly to remove dust and soil particles, air-dried at room temperature for two weeks, and subsequently pulverized into a fine powder using a sterile mechanical grinder. The powdered sample was stored in airtight containers until extraction [40]. The use of *Curcuma longa* as a medicinal plant is well documented due to its rich phytochemical composition and broad pharmacological relevance in cardiovascular and cancer-related pathways [7,11].

### 4.2. Extraction Procedure

A weighed quantity of the powdered rhizome was extracted using absolute ethanol for 24 h under continuous agitation. The mixture was filtered, and the filtrate was concentrated by solvent evaporation under reduced pressure. The crude extract obtained was stored for subsequent GC–MS and computational analyses. Ethanolic extraction is widely applied in natural product drug discovery due to its efficiency in recovering both polar and semi-polar bioactive compounds [6,30].

### 4.3. Gas Chromatography–Mass Spectrometry (GC–MS) Analysis

Phytochemical profiling was conducted using a GC–MS system (Agilent Technologies 7890 GC and 5977B MSD, Agilent Technologies, Santa Clara, CA, USA). Separation was achieved using an HP-5 MS capillary column (30 m × 0.25 mm internal diameter, 0.25 µm film thickness). Helium was used as the carrier gas at a constant flow rate of 1.0 mL/min. The oven temperature was programmed from 40 °C to 250 °C at a rate of 50 °C/min. The injection volume was 1 µL, and samples were analyzed within a mass range of 40–650 *m*/*z*. Compound identification was performed using the National Institute of Standards and Technology (NIST) mass spectral library. GC–MS-based metabolite profiling remains a standard analytical technique in phytochemical-driven computational drug discovery workflows [41].

### 4.4. Ligand Preparation

Identified compounds from GC–MS analysis were retrieved in 3D structure format from the PubChem database. Structural optimization was performed using Open Babel (Python Prescription v0.8) with the MMFF94 force field to obtain energetically stable conformations. Ligand preparation followed standard computer-aided drug design (CADD) protocols commonly used in natural product screening [20,35].

### 4.5. Protein Target Selection and Preparation

Protein targets associated with cardiovascular and cancer pathways were selected based on their biological relevance: HMG-CoA reductase (cholesterol biosynthesis and CVD pathway), PI3Kγ (cardiovascular signaling and thrombosis pathway), CDK6 (cell cycle regulation in cancer progression), and HER2 kinase domain (breast cancer signaling pathway). The 3D crystal structures were retrieved from the Protein Data Bank (PDB): IT02 (HMG-CoA reductase–statin complex), 4URK (PI3Kγ–AZD6482 complex), 4EZ5 (CDK6–inhibitor complex), 3RCD (HER2–TAK-285 complex) Protein preparation involved removal of co-crystallized ligands and water molecules, addition of hydrogen atoms, and energy minimization using Cresset Flare^®^ (v4.0) with the General Amber Force Field (GAFF). Minimization parameters were set to a 0.200 kcal/mol/Å gradient cut-off with 2000 iterations. This protocol is consistent with modern molecular docking preparation standards used in recent cancer and cardiovascular drug discovery studies [21,23,35,42].

### 4.6. Molecular Docking Studies

Molecular docking was performed using AutoDock Vina integrated into Python Prescription 0.8 and validated through PyRx-based workflows (PyRx-Python prescription 0.8). Flexible docking was applied to evaluate ligand–protein interactions. Grid box parameters were individually defined for each target protein to ensure correct active site coverage. Binding affinities were calculated in kcal/mol, and docking poses were analyzed based on hydrogen bonding, hydrophobic interactions, and π–π stacking interactions. Docking validation was performed by re-docking co-crystallized ligands. This approach follows established methodologies for curcumin and phytochemical-based in silico drug screening [6,25,26].

### 4.7. PASS Prediction (Biological Activity Spectrum Analysis)

The biological activity spectrum of selected phytochemicals was predicted using PASS Online way2Drug.com. ©2011-2026 version 2.0 (Prediction of Activity Spectra for Substances). This method estimates the probability of pharmacological activity based on structural similarity to known active compounds. PASS analysis has been widely used in natural product-based drug discovery to identify anticancer and cardioprotective leads [33].

### 4.8. ADMET and Drug-likeness Evaluation

Pharmacokinetic properties (Absorption, Distribution, Metabolism, Excretion, and Toxicity—ADMET) were evaluated using the SwissADME web tool by the molecular modelling group of the SIB(Swiss institute of Bioinformatics). Drug-likeness was assessed using Lipinski’s Rule of Five, bioavailability score, and physicochemical properties, including solubility and gastrointestinal absorption. ADMET prediction is an essential early-stage filter in modern drug discovery pipelines for identifying clinically viable lead compounds [27,28,43].

### 4.9. Density Functional Theory (DFT) Calculations

Quantum chemical calculations were performed using the DMol^3^ module in BIOVIA Materials Studio 8.0. Geometry optimization was carried out using the Local Density Approximation (LDA) with the Perdew–Wang correlation functional (PWC). The Double Numerical plus Polarization (DNP) basis set was applied. Convergence criteria were set as follows:

Energy: 2 × 10^−5^ Ha

Maximum force: 0.004 Ha/Å

Displacement: 5 × 10^−3^ Å

Maximum iterations: 50

Frontier molecular orbital parameters (HOMO, LUMO) were calculated to determine chemical reactivity and stability:

(1) $$E_{gap}=E_{LUMO}-E_{HUMO}$$

(2) $$\mu =-\chi =\frac{E_{HUMO}-E_{LUMO}}{2}$$

IE = −E_HOMO_ (3)

EA = −E_LUMO_ (4)

Global hardness and softness were calculated according to Parr and Pearson theory [44,45].

(5) $$n=\frac{IE-EA}{2}$$

(6) $$s=\frac{1}{n}$$

DFT-based molecular evaluation is widely used to complement docking studies in phytochemical drug discovery [35,38].

### 4.10. Statistical and Multivariate Data Analysis

Descriptive statistics were performed using Microsoft Excel 2016. Multivariate analysis, including Principal Component Analysis (PCA), was conducted using OriginLab Pro 8 and SPSS version 23. Varimax rotation was applied, and components with eigenvalues greater than 1 were retained according to the Kaiser criterion. Linear regression analysis was used to determine relationships between binding affinities and molecular descriptors. Multivariate statistical approaches are commonly used in computational pharmacology to correlate structural and biological activity trends [36,41].

## 5. Conclusions

This study provides an integrated in silico and phytochemical evaluation of *Curcuma longa* L. rhizome, combining GC–MS profiling, molecular docking, PASS prediction, ADMET analysis, density functional theory (DFT), and statistical modeling to explore its cardioprotective and anticancer potential.

GC–MS analysis confirmed that *Curcuma longa* is rich in bioactive sesquiterpenes, particularly curlone, turmerone, curcumene, and related derivatives, which are known for their broad pharmacological relevance. Molecular docking results demonstrated that several of these compounds exhibit strong and stable binding interactions with key cardiovascular and cancer-related targets, including HMG-CoA reductase, PI3Kγ, CDK6, and HER2 kinase. Among all identified compounds, curlone and turmerone consistently showed the most favorable binding affinities and multi-target interaction profiles.

PASS prediction further supported their broad pharmacological potential, indicating high probabilities for anticancer, anti-inflammatory, antioxidant, and cardioprotective activities. In addition, ADMET evaluation suggested that the major active compounds possess acceptable drug-likeness and pharmacokinetic properties, supporting their suitability as lead-like candidates. DFT analysis confirmed their electronic stability and reactivity profiles, reinforcing their ability to form stable ligand–protein interactions.

Overall, the convergence of docking, pharmacokinetic, quantum chemical, and statistical analyses highlights *Curcuma longa* as a promising natural source of multi-target bioactive compounds with potential therapeutic relevance in both cardiovascular diseases and cancer. In particular, curlone and turmerone emerge as leading candidates for further development.

However, these findings are based on computational predictions and require experimental validation through in vitro enzymatic assays, cell-based studies, and in vivo models to confirm their biological efficacy and safety.

In conclusion, this work supports the concept that *Curcuma longa* phytochemicals may serve as valuable scaffolds for the development of novel multi-target therapeutic agents targeting interconnected pathways in cardiovascular disease and cancer.

## Figures and Tables

**Table 1:** Result of the molecular docking for cardioprotective activity against (1T02) and (4URK) proteins.

Compounds Numbers	Binding Affinity (kcal/mol) Against 1T02	Binding Affinity (kcal/mol) Against 4URK
**1**	−5.0	−6.7
**2**	−4.8	−6.1
**3**	−5.0	−6.1
**4**	−6.3	−6.3
**5**	−4.7	−5.7
**6**	−5.9	−7.6
**7**	−5.7	−7.0
**8**	−6.1	−7.4
**9**	−5.5	−7.4
**10**	−6.4	−7.6
**11**	−6.4	−7.1
**12**	−6.0	−7.2
**13**	−3.7	−4.5
**14**	−4.0	−4.5
**15**	−4.6	−6.3
**16**	−5.2	−5
**17**	−5.4	−5.6
Cocrystal ligand for 1T02 Lovastatin acid	−6.0	−7.7
Drug lovastatin Anticholesteremic and antineoplastic agent	−7.2	−8.1
Atorvastatin Anticholesteremic Drug	−7.1	−7.6
Cocrystal ligand of 4URK protein	−7.1	−10
Clopidogrel Platelet drug	−5.6	−6.9
Aspirin platelet and multi-function drugs	−4.9	−5.6

**Key to compounds names:** Compound **1**: 12-Methyl-E,E-2,13-octadecadien-1-ol; Compound **2**: o-Cymene; Compound **3**: Cyclopropyl phenylmethanol; Compound **4**: Caryophyllene; Compound **5**: 2,6-Octadienal, 3,7-dimethyl-, (Z); Compound **6**: Benzene, 1-(1,5-dimethyl-4-hexenyl)-4-methyl- or alpha curcumene; Compound **7**: Bicyclo[3.1.1]heptane,6methyl-2-methylene-6-(4-methyl-3-pentenyl)-[1R-(1.alpha.,5.alpha.,6.beta.)] or bergamotene; Compound **8**: Cyclohexene, 3-(1,5-dimethyl-4-hexenyl)-6-methylene-, [S-(R*,S*)]-; Compound **9**: Benzene, 1-(1,5-dimethylhexyl)-4-methyl-; Compound **10**: Tumerone; Compound **11**: Curlone; Compound **12**: (6R,7R)-Bisabolone; Compound **13**: 2-Butenoic acid, 3-methyl-, methylester; Compound **14**: 2-Pyrazoline, 1-allyl; Compound **15**: 8-Hexadecenal, 14-methyl-, (Z)-; Compound **16**: 2-tert-Butyl-5,5-dimethyl-3-oxo-1- pyrroline, 1-oxide; Compound **17**: Cyclooctaneacetic acid, 2-oxo-.

**Table 2:** Result of the molecular docking for anticancer activity against (4EZ5) and (3RCD) proteins.

Compounds	Binding Affinity Against 4EZ5	Binding Affinity Against 3RCD
**1**	−6.3	−6.2
**2**	−6.3	−5.7
**3**	−6.6	−6
**4**	−8	−6.5
**5**	−6	−5.4
**6**	−8.2	−6.7
**7**	−7.5	−6.9
**8**	−7.4	−7.0
**9**	−7.7	−6.7
**10**	−7.6	−7.1
**11**	−7.5	−7.2
**12**	−7.8	−7.1
**13**	−4.6	−4.4
**14**	−4.5	−4.1
**15**	−6.1	−5.8
**16**	−6.5	−6.4
Cyclooctaneacetic acid17, 2-oxo-	−6.4	−6.2
Cocrystalline ligand of 4EZ5 [5-[4-(dimethylamino)piperidin-1-yl]-1*H*-imidazo [4,5-b]pyridin-2-yl]-(2-isoquinolin-4-ylpyridin-4-yl)methanone	−11.3	−11.5
Standard cancer Drug Abemaciclib, a CDK 6/4 inhibitor	−9.8	−9.9
Palbociclib	−10	9.6
Cocrystalline ligand of 3RCD TAK-285	−9.6	−9.7
Drug lapatinib drug against HER2 and EGFR	−9.9	−10.1
Drug Afatinib	−9.3	−9.2

**Table 3:** Result of pharmacokinetic (ADMET) properties and Drugability of the good compounds.

**Compounds**								
**Models**	**A**	**B**	**C**	**D**	**E**	**F**	**G**	**H**
**Physiochemical parameter**								
Weight	202.3	204.35	204.35	204.35	204.35	218.33	218.33	220.35
Rotable bond	4	0	3	4	5	4	4	4
HBA	0	0	0	0	0	1	1	1
HBD	0	0	0	0	0	0	0	0
Molar Refractivity	69.55	68.78	68.78	70.68	70.02	70.88	70.88	71.36
TPSA	0	0	0	0	0	17.07	17.07	17.07
ILOG P	3.5	3.29	3.52	3.58	3.59	3.19	3.14	3.34
XLOG P3	5.38	4.38	6.13	5.41	5.83	3.33	4.01	4.82
WLOG P	4.84	4.73	4.73	4.89	4.92	4.21	4.07	4.29
MLOG P	5.75	4.63	4.63	4.53	5.84	3.37	3.37	3.46
**Pharmacokinetic (ADMET)**								
Consensus Log P	4.86	4.24	4.71	4.56	5.03	3.63	3.7	4.02
Log S (Water Solubility)	−4.52	−3.87	−4.77	−4.25	−4.75	−3.63	−3.46	−3.98
GI Absorption	Low	Low	Low	Low	Low	High	High	High
BBB Permeant	No	No	No	No	No	Yes	Yes	Yes
P-gp Substrate	No	No	No	No	No	No	No	No
CYP 1A2 Inhibitor	No	No	No	No	No	No	No	No
CYP 2C19 Inhibitor	No	Yes	Yes	Yes	No	No	Yes	Yes
CYP 2C9 Inhibitor	No	Yes	Yes	Yes	No	Yes	Yes	Yes
CYP 2D6 Inhibitor	Yes	No	No	No	Yes	No	No	No
CYP 3A4 Inhibitor	No	No	No	No	No	No	No	No
Log Kp (Skin Permeation) cm/s	−3.71	−4.44	−3.19	−3.71	−3.41	−5.27	−4.78	−4.22
**Drugability**								
Lipinski	Yes (1)	Yes (1)	Yes (1)	Yes (1)	Yes (1)	Yes (0)	Yes (0)	Yes (0)
Ghose	Yes	Yes	Yes	Yes	Yes	Yes	Yes	Yes
Veber	Yes	Yes	Yes	Yes	Yes	Yes	Yes	Yes
Egan	Yes	Yes	Yes	Yes	Yes	Yes	Yes	Yes
Muegge	No	No	No	No	No	No	No	No
Bioavailability	0.55	0.55	0.55	0.55	0.55	0.55	0.55	0.55
**Medicinal Chemistry**								
PAINS	0 alert	0 alert	0 alert	0 alert	0 alert	0 alert	0 alert	0 alert
Brenk	1 alert	1 alert	1 alert	1 alert	0 alert	1 alert	1 alert	1 alert
LeadLikeness	No	No	No	No	No	No	No	No
Synthetic Accessibility	2.31	4.51	4.68	4.42	2.02	4.3	4.17	3.91

A = Curcumene; B = Caryophyllene; C = bergamotene; D = Cyclohexene, 3-(1,5-dimethyl-4-hexenyl)-6-methylene-, [S-(R*,S*)]-; E = Benzene, 1-(1,5-dimethylhexyl)-4-methyl-; F = Tumerone; G = Curlone; H = (6R,7R)-Bisabolone.

**Table 4:** Result of pharmacokinetic (ADMET) properties and Drugability of the Cocrystalline ligands and the standard drugs.

**Compounds**											
**Models**	**M**	**N**	**O**	**P**	**Q**	**R**	**S**	**T**	**U**	**V**	**W**
**Physical properties**											
Weight	422.5	404.5	558.6	409	322	180	478	507	448	548	486
Rotable bond	11	7	13	5	4	3	5	7	5	11	9
HBA	6	5	6	5	3	4	6	8	6	8	7
HBD	3	1	4	2	0	1	1	1	2	3	2
Molar Refractivity	117.7	113.9	158.3	118	88.9	45	144	149	136	138	130
TPSA	104.1	72.8	111.8	96.2	57.8	64	90.9	75	105	101	88.6
ILOGP	3.19	3.94	3.81	2.9	3.36	1.3	3.35	4.16	3.39	3.79	4.27
XLOGP	4.04	4.26	4.96	1.96	3.75	1.2	4.25	3.84	1.81	4.4	3.64
WLOP	3.72	4.2	6.54	1.81	2.82	1.3	3.95	4.86	2.2	7.07	4.62
MLOGP	2.75	3.51	3.48	0.6	2.93	1.5	1.58	3.04	2.03	2.52	2.3
**Pharmacokinetic (ADMET)**											
Consensus Log P	3.37	3.89	4.99	1.88	3.5	1.3	3.43	4.04	2.39	4.48	3.73
Log S (Water Solubility)	−4.28	−4.57	−5.99	−3.7	−4.32	−1.9	−5.66	−5.36	−3.8	−5.69	−4.9
GI Absorption	High	High	Low	High	High	High	High	High	High	Low	High
BBB Permeant	No	Yes	No	No	Yes	Yes	No	No	No	No	No
P-gp Substrate	Yes	No	Yes	Yes	No	No	Yes	Yes	Yes	Yes	Yes
CYP 1A2 Inhibitor	No	No	No	No	No	No	No	No	No	Yes	No
CYP 2C19 Inhibitor	No	No	Yes	No	Yes	No	Yes	Yes	No	Yes	Yes
CYP 2C9 Inhibitor	No	Yes	No	Yes	Yes	No	Yes	Yes	Yes	Yes	Yes
CYP 2D6 Inhibitor	No	No	Yes	No	Yes	No	Yes	Yes	Yes	Yes	Yes
CYP 3A4 Inhibitor	Yes	Yes	Yes	Yes	No	No	No	Yes	Yes	Yes	Yes
Log Kp	−6.01	−5.74	−6.19	−7.4	−5.6	−6.6	−6.2	−6.66	−7.7	−6.52	−6.7
**Drugability (Druglieness)**											
Lipinski	Yes (0)	Yes (0)	Yes (1)	Yes (0)	Yes (0)	Yes (0)	Yes (0)	Yes (1)	Yes (0)	Yes (1)	Yes (0)
Ghose	Yes	Yes	No (4)	Yes	Yes	Yes	No (1)	No (2)	No (1)	No (3)	No (1)
Veber	No (1)	Yes	No (1)	Yes	Yes	Yes	Yes	Yes	Yes	No (1)	Yes
Egan	Yes	Yes	No (1)	Yes	Yes	Yes	Yes	Yes	Yes	No (1)	Yes
Muegge	Yes	Yes	Yes	Yes	Yes	No (1)	Yes	Yes	Yes	Yes	Yes
Bioavaliability	0.56	0.55	0.56	0.56	0.55	0.9	0.55	0.55	0.55	0.55	0.55
**Medicinal Chemistry**											
PAINS	0 alert	0 alert	0 alert	0 alert	0 alert	0 alert	0 alert	0 alert	0 alert	0 alert	0 alert
Brenk	0 alert	1 alert	0 alert	0 alert	0 alert	1 alert	0 alert	0 alert	0 alert	0 alert	1 alert
LeadLikeness	No (3)	No (2)	No (3)	No (1)	No (1)	No (1)	No (2)	No (2)	No (1)	No (3)	No (3)
Synthetic	5.89	5.76	4.95	3.6	3.21	1.5	3.72	3.87	3.57	3.71	4.08

M = Lovastatin acid; N = Lovastatin; O = Atorvastatin; P = Cocrystalline AZD6482; Q = Clopidogrel; R = Aspirin; S = Cocrystalline ligand of 4EZ5 [5-[4-(dimethylamino)piperidin-1-yl]-1*H*-imidazo [4,5-b]pyridin-2-yl]-(2-isoquinolin-4-ylpyridin-4-yl)methanone; T = Abemaciclib; U = Palbociclib; V = Laptinib; W = Afatinib.

**Table 5:** Quantum chemical parameters of bioactive compounds of *Curcuma longa* L. root.

Ligands	E_T_ (Ha)	BE (Ha)	HOMO (eV)	LUMO (eV)	Energy Gap (eV)	µ (eV)	X (eV)	IE (eV)	EA (eV)	n (eV)	s (eV)
12-Methyl-E,E-2,13-octadecadien-1-ol	−812.61	−9.05	−3.68	−3.50	0.18	−0.09	0.09	3.68	3.50	0.09	10.99
o-Cymene	−385.66	−4.32	−4.41	−4.12	0.29	−0.14	0.14	4.41	4.12	0.14	6.97
Cyclopropyl phenylmethanol	−459.47	−4.56	−5.84	−0.98	4.86	−2.43	2.43	5.84	0.98	2.43	0.41
Caryophyllene	−579.49	−6.05	−3.81	−3.20	0.61	−0.30	0.30	3.81	3.20	0.30	3.28
2,6-Octadienal, 3,7-dimethyl-, (Z)	−461.55	−4.73	−4.93	−4.41	0.52	−0.26	0.26	4.93	4.41	0.26	3.82
Benzene, 1-(1,5-dimethyl-4-hexenyl)-4-methyl- or alpha curcumene	−579.05	−6.56	−4.29	−4.07	0.23	−0.11	0.11	4.29	4.07	0.11	8.85
Bicyclo[3.1.1]heptane,6methyl-2-methylene-6-(4-methyl-3-pentenyl)-[1R-(1.alpha.,5.alpha.,6.beta.)] or bergamotene	−580.18	−6.73	−4.71	−4.42	0.29	−0.15	0.15	4.71	4.42	0.15	6.87
Cyclohexene, 3-(1,5-dimethyl-4-hexenyl)-6-methylene-, [S-(R*,S*)]-	−580.15	−6.70	−3.56	−3.52	0.05	−0.02	0.02	3.56	3.52	0.02	42.55
Benzene, 1-(1,5-dimethylhexyl)-4-methyl-	−580.47	−7.02	−3.94	−3.03	0.91	−0.45	0.45	3.94	3.03	0.45	2.21
Tumerone	−653.51	−6.50	−3.17	−1.85	1.32	−0.66	0.66	3.17	1.85	0.66	1.51
Curlone	−653.57	−6.56	−3.19	−1.93	1.26	−0.63	0.63	3.19	1.93	0.63	1.58
(6R,7R)-Bisabolone	−654.72	−6.75	−3.26	−2.95	0.31	−0.16	0.16	3.26	2.95	0.16	6.43
2-Butenoic acid, 3-methyl-, methylester	−381.61	−2.99	−4.21	−3.96	0.26	−0.13	0.13	4.21	3.96	0.13	7.84
2-Pyrazoline, 1-allyl	−341.04	−3.20	−3.08	−0.94	2.15	−1.07	1.07	3.08	0.94	1.07	0.93
8-Hexadecenal, 14-methyl-, (Z)-	−734.85	−8.13	−5.17	−4.00	1.17	−0.59	0.59	5.17	4.00	0.59	1.70
2-tert-Butyl-5,5-dimethyl-3-oxo-1- pyrroline, 1-oxide	−581.29	−3.45	−6.10	−5.38	0.73	−0.36	0.36	6.10	5.38	0.36	2.76
Cyclooctaneacetic acid,											

E_T_: Total energy; BE: Binding energy; HOMO: highest occupied molecular orbital; LUMO: lowest unoccupied molecular orbital; µ: Electronic chemical potential; X: electronegativities; IE: ionization potential; EA: electron affinity; n: global hardness; s: global softness.

## Data Availability

The original contributions presented in this study are included in the article/Supplementary Material. Further inquiries can be directed to the corresponding author.

## Supplementary Materials

The following supporting information can be downloaded at: https://www.mdpi.com/article/10.3390/ph19060834/s1, Table S1: Result of the GC-MS analysis, Table S2: Represent the table for cocrystalline ligands of the different proteins that was used for the docking and some standard cardioprotective and anticancer drugs, Table S3: Interaction of the best compounds from turmeric, drugs and cocrystalline ligand against the CVD disease protein and its amino acids receptors, Table S4: Interaction of the best compounds from turmeric, drugs and cocrystalline ligand against the cancer disease protein and its amino acids receptors, Table S5: Biological activities of Curcumene, Table S6: Biological activities of Caryophyllene, Table S7: Biological activities of Bergamotene, Table S8: Biological activities of Cyclohexene, 3-(1,5-dimethyl-4-hexenyl)-6-methylene-, [S-(R*,S*)]-, Table S9: Biological activities of Benzene, 1-(1,5-dimethylhexyl)-4-methyl-, Table S10: Biological activities of Tumerone, Table S11: Biological activities of Curlone, Table S12: Biological activities of (6R,7R)-Bisabolone.
