# Supplementary material for: Assessment and Density Functional Theory of Bioactive Compounds of Curcuma longa L. Root Responsible for Its Cardio-Protective and Anti-Cancer Activities
Source: Pharmaceuticals (Basel). 2026 May 27;19(6):834. doi: 10.3390/ph19060834 (PMC13304733; doi:10.3390/ph19060834)
Supplement: Supplementary file 1 [file pharmaceuticals-19-00834-s001.zip › pharmaceuticals-4321553-supplementary.pdf]

Table S1: Result of the GC-MS analysis of Turmeric

S/N	RT	Name of Compound	PubChem ID	Percentage composition	Molecular Formular	Structure
1	7.57	12-Methyl-E,E-2,13-octadecadien-1-ol	90107969	6.11	$C_{19}H_{36}O$	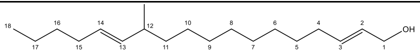
2	8.14	o-Cymene	10703	5.44	$C_{10}H_{14}$	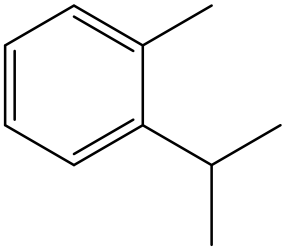
3	8.68	Cyclopropyl phenylmethanol	66090	1.67	$C_{19}H_{36}O$	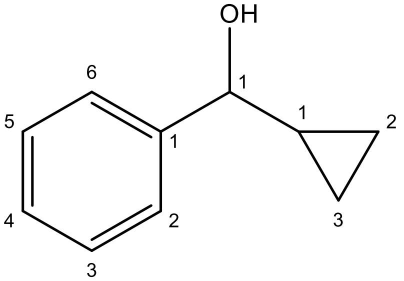
4	9.02	Caryophyllene	5281515	5.61	$C_{19}H_{36}O$	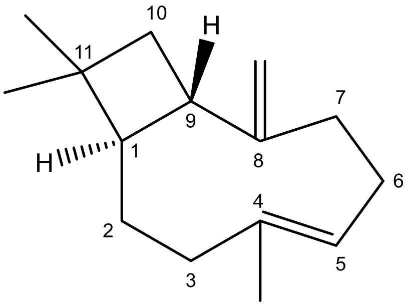
5	9.65	2,6-Octadienal, 3,7-dimethyl-, (Z)	638011	3.28	$C_{10}H_{16}O$	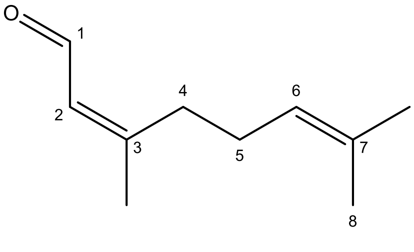
6	10.60	Benzene, 1-(1,5-dimethyl-4-hexenyl)-4-methyl- or alpha curcumene	92139	2.17	$C_{15}H_{24}$	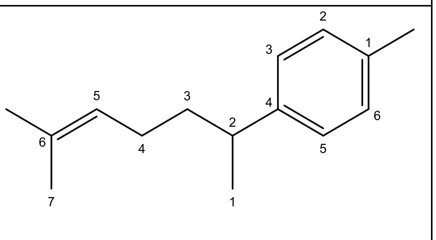

7	10.95	Bicyclo[3.1.1]heptane,6methyl-2-methylene-6-(4-methyl-3-pentenyl)-[1R-(1.alpha.,5.alpha.,6.beta.)] or bergamotene	521569	1.66	$C_{15}H_{24}$	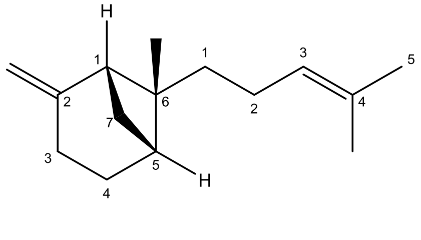
8	11.60	Cyclohexene, 3-(1,5-dimethyl-4-hexenyl)-6-methylene-, [S-(R*,S*)]-	519764	0.97	$C_{15}H_{24}$	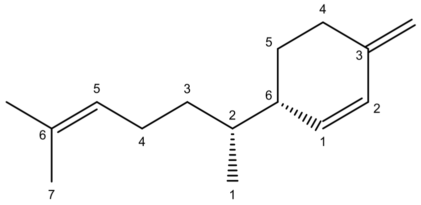
9	13.06	Benzene, 1-(1,5-dimethylhexyl)-4-methyl-	577053	0.28	$C_{15}H_{24}$	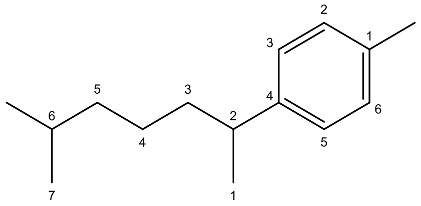
10	14.99	Tumerone	558173	13.85	$C_{15}H_{22}O$	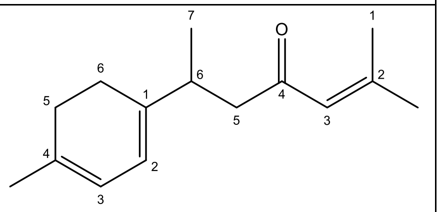
11	15.71	Curlone	196216	20.99	$C_{15}H_{22}O$	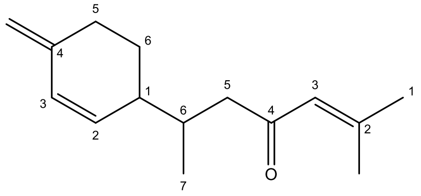
12	16.49	(6R,7R)-Bisabolone	11321983	0.79	$C_{15}H_{24}O$	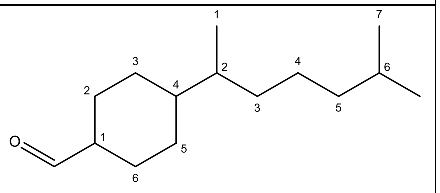
13	16.717	2-Butenoic acid, 3-methyl-, methylester	13546	4.1	$C_6H_{10}O_2$	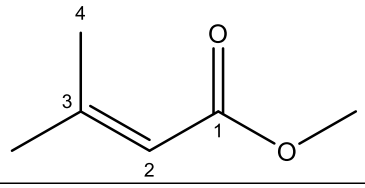

14	19.82	2-Pyrazoline, 1-allyl	557756	0.59	$C_6H_{10}N_2$	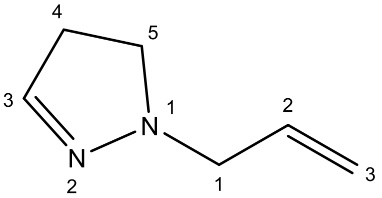
15	25.00	8-Hexadecenal, 14-methyl-, (Z)-	5364688	0.19	$C_{17}H_{32}O$	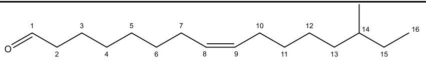
16	31.20	2-tert-Butyl-5,5-dimethyl-3-oxo-1-pyrroline, 1-oxide	249899402	1.41	$C_{10}H_{17}NO_2$	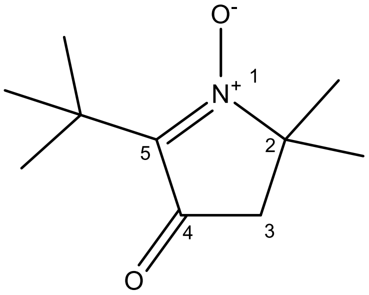
17	32.70	Cyclooctaneacetic acid, 2-oxo-	536995	30.88	$C_{10}H_{16}O_3$	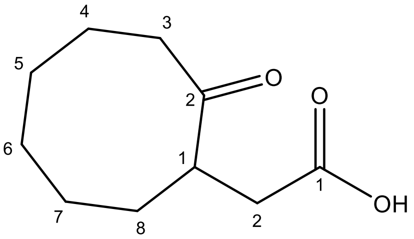

Table S2: represent the table for cocrystalline ligands of the different proteins that was used for the docking and some standard cardioprotective and anticancer drugs

SN	Cocrystalline ligands/Drugs	PubChem ID	Structures
1	Cocrystal ligand for 1T02<br>Lovastatin acid	64727	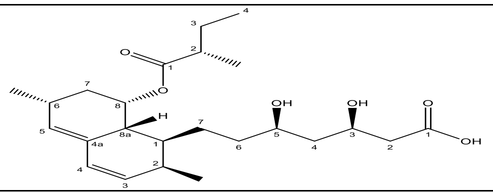
2	Drug lovastatin<br>Anticholesteremic and antineoplastic agent	53232	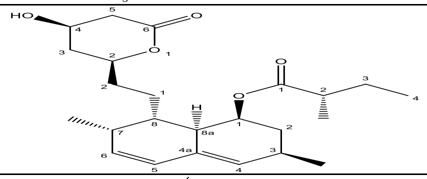
3	Atorvastatin<br>Anticholesteremic drug	60823	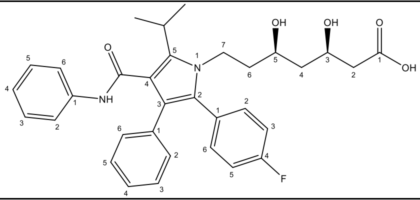

4	Cocrystal ligand of 4URK protein	44137675	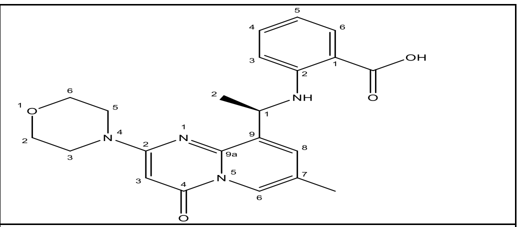
5	Clopidogrel Platelet drug	60606	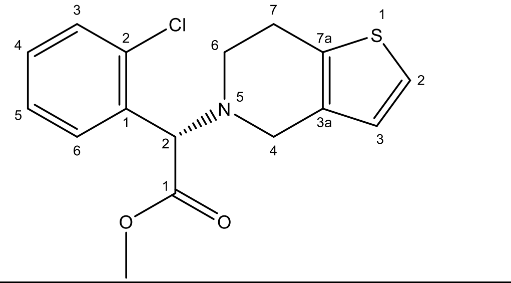
6	Aspirin platelet and multi-function drugs	2244	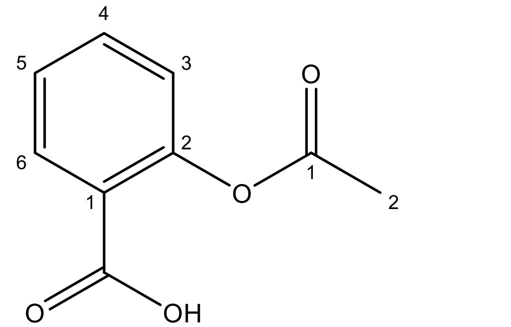
7	Cocrystalline ligand of 4EZ5 [5-[4-(dimethylamino)piperidin-1-yl]-1*H*-imidazo[4,5-b]pyridin-2-yl]-(2-isoquinolin-4-ylpyridin-4-yl)methanone	49785477	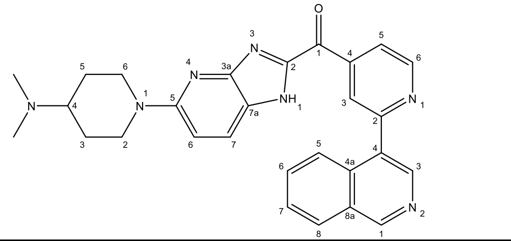
8	Standard cancer Drug Abemaciclib CDK 6/4 inhibitor	46220502	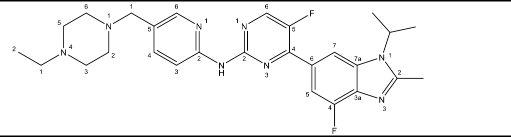
9	palbociclib	5330286	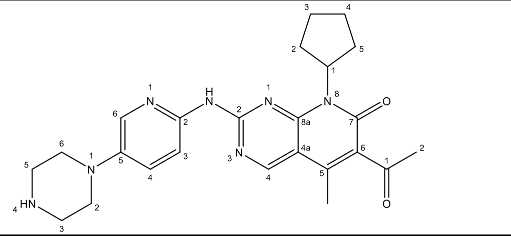

10	Cocrystalline ligand of 3RCD TAK-285	11620908	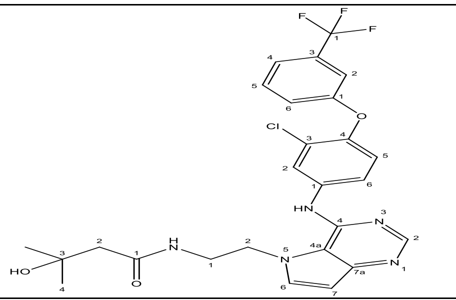
11	Drug lapatinib drug against HER2 and EGFR	208908	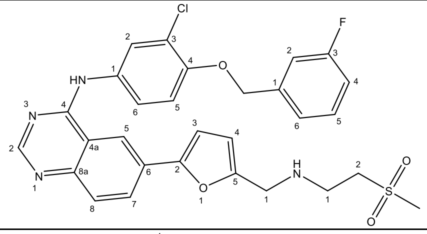
12	Drug Afatinib	10184653	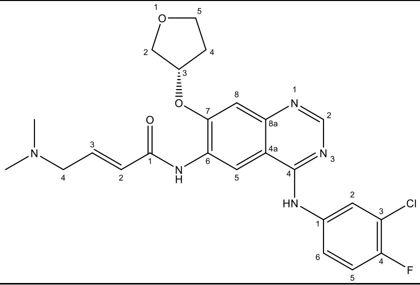

Table S3: Interaction of the best compounds from turmeric, drugs and cocrystalline ligand against the CVD disease protein and its amino acids receptors.

**Interaction for 1T02**		
**Compounds**	**Type of interaction**	**Receptor/ Amino acid**
Lovastatin Drug	Hydrogen bonding	TRP 284
	Alkyl	ARG 285, LYS 267
Cocrystalline ligand	Hydrogen bonding	ASP 274, TRP 284, GLU 288
	Alkyl/Pi-Alkyl	VAL 81
Curlone	Alkyl/Pi-Alkyl	LEU 60, LYS 267, MET 270, VAL 278, TRP 284
Cyclohexene, 3-(1,5-dimethyl-4-hexenyl)-6-methylene-, [S-(R*,S	Alkyl/Pi-Alkyl	LYS 267, VAL 278, 284
Tumerone	Alkyl/Pi-Alkyl	LEU 60, LYS 267, MET 270, VAL 278, TRP 284
Caryophyllene	Alkyl/Pi-Alkyl	TPR 284
Bisabolone	Alkyl/Pi-Alkyl	LEU 60, VAL 81, LYS 267, MET 270, PRO 275, VAL 278
	Pi-Sigma	TRP 284
**Interaction of 4URK**		
**Compounds**	**Type of interaction**	**Receptor/ Amino acid**
Aspirin (Drug)	Carbon-Hydrogen	ILE 881
	Pi-sigma	TYP 867, ILE 963,

Clopidogrel (Drug)	Hydrogen Bonding	VAL 882
	Pi-Sulfur	MET 804, PHE 961
	Pi-Pi -T- Shaped	TYR 867
	Alkyl/Pi-Alkyl	PRO 810, LYS 833, ILE 879, ILE 831, ILE 963, MET 953
Curcumene	Pi-Pi -T- Shaped	TYR 867
	Pi-Sigma	ILE 963
	Alkyl/Pi-Alkyl	VAL 882, PHE 961, MET 953, ILE 879, PRO 810, MET 804, ILE 831
Curlone	Hydrogen Bonding	ASP 964
	Pi-sigma	TYR 867
	Alkyl/Pi-Alkyl	ILE 831, PRO 810, MET 804, ILE 963, ILE 879
Cyclohexene	Pi-sigma	TYP 867
	Alkyl/Pi-Alkyl	VAL 882, MET 953, ILE 963, ILE 879, PHE 961, PRO 810, MET 804, ILE 831
Tumerone	Hydrogen Bonding	ASP 964
	Pi-sigma	TYR 867
	Alkyl/Pi-Alkyl	ILE 963, ILE 879, PHE 961, MET 804, ILE 831, PRO 810, TRP 812
Bisabolone	Hydrogen Bonding	ASP 964
	Pi-Sigma	TPR 867
	Alkyl/Pi-Alkyl	ILE 831, PRO 810, LYS 833, ILE 879, TRP 812, ILE 963, PHE 961
Cocrystal ligand of 4URK	Carbon-Hydrogen	GLU 880
	Pi-sigma	TRP 812, ILE 963
	Pi-Sulfur	MET 804
	Alkyl/Pi-Alkyl	ILE 879, PRO 810, ILE 831

Table S4: Interaction of the best compounds from turmeric, drugs and cocrystalline ligand against the cancer disease protein and its amino acids receptors.

**Interaction for 4EZ5**		
**Compound**	**Type of interaction**	**Receptor/Amino acid**
Curcumene	Pi-Sigma	PHE 164
	Pi-Pi -T-Shape	PHE 98
	Alkyl-Pi-alkyl	LEU 65, ALA 41, ALA 162, VAL77, LEU 152, ILE 19
Curlone	Hydrogen bond	LYS 43
	Pi-Sigma	PHE 98
	Alkyl-Pi-alkyl	ALA 41, ALA 162, VAL 27
Bergamotene	Pi-Sigma	PHE 98
	Alkyl-Pi-alkyl	VAL 27, ILE 19, ALA 162, VAL 77, ALA 41, LEU 152
Tumerone	Pi-Sigma	PHE 98
	Alkyl-Pi-alkyl	ILE 19, LEU 152, VAL 77, ALA 162

Benzene, 1-(1,5-dimethylhexyl)-4-methyl-	Pi-Sigma	PHE 98,
	Alkyl-Pi-alkyl	ALA 41,VAL 77, ALA 162
Caryophyllene	Pi-Sigma	PHE 98
	Alkyl-Pi-alkyl	ALA 162, ILE 19, VAL 27
Palbociclib	Hydrogen bond	VAL 101, ASP 163
	Carbon-Hydrogen bond	ASP 104, GLU 99
	Alkyl-Pi-alkyl	ILE 19, VAL 27, ALA 41, LEU 152, ALA 162
Bisabolone	Pi-Sigma	PHE 98
	Alkyl-Pi-alkyl	ILE 19, LEU 152, VAL 77, ALA 41, ALA 162
	Hydrogen bond	VAL 101, ASP 104, GLN 149
Abemacicib	Carbon-Hydrogen bond	GLU 21, ASP 163, HIS 100, ALA 23
	Pi-Sigma	ILE 19, LEU 152
	Halogen	GLU 99, GLN 103
	Alkyl-Pi-alkyl	ALA 41, ALA 162
Cocrystalliine of 4EZ5	Hydrogen bond	VAL 101, GLU 99, LYS 43
	Pi-donor Carbon-Hydrogen	ALA 23, ASP 145, GLU 21, HIS 100
	Pi-Anion	ASP 163
	Pi-Sigma	LEU 152, ILE 19
	Alkyl-Pi-alkyl	VAL 27, ALA 162
**Interaction of 3RCD**		
Curcumene	Pi-Sigma	LEU 785
	Alkyl-Pi-alkyl	LYS 753, LEU 796, ALA 751, VAL 734, MET 774, PHE 864
Curlone	Hydrogen bond	SER 783
	Alkyl-Pi-alkyl	LEU 852, LYS 753, VAL 734, ALA 751, PHE 864, MET 774, LEU 785
Lapatinib	Hydrogen bond	SER 783
	Caron-hydrogen bond	ASP 863, GLN 799
	Halogen	ARG 784
	Unfavourable D-D	MET 801
	Pi-Sigma	LEU 785, LEU 726
	Pi-Sulfur	MET 801
	Alkyl-Pi-alkyl	LYS 753, LEU 852, ALA 751
Cyclohexene	Alkyl-Pi-alkyl	VAL 734, LEU 852, LEU 726, MET 801, LEU 852, PHE 1004, LYS 753, LEU 796
Bergamotene	Alkyl-Pi-alkyl	ALA 751, VAL 734, LEU 800, MET 801, LEU 852, PHE 1004, LYS 753
Tumerone	Hydrogen bond	ASP 863, THR 862
	Alkyl-Pi-alkyl	MET 774, LEU 785, LEU 796, PHE 864, VAL 734, LYS 753, ALA 751, LEU 852
Bezene-	Pi-Sigma	LYS 753,

	Alkyl-Pi-alkyl	LEU 785, LEU 796, ALA 751, VAL 734, LEU 852
Afatinib	Hydrogen bond	ALA 751, LYS 753
	Caron-hydrogen bond	LEU 796
	Alkyl-Pi-alkyl	VAL 734, PHE 731
Bisabolone	Hydrogen bond	SER 783
	Unfavourable A-A	THR 862
	Alkyl-Pi-alkyl	PHE 864, MET 774, LYS 753, VAL 734, LEU 852, ALA 751
Tak-285	Halogen	ARG 784
	Unfavourable D-D	MET 801
	Pi-Pi-T-Shape	PHE 864
	Pi-Sigma	LEU 785
	Caron-hydrogen bond	GLN 799
	Hydrogen bond	SER 783, ASP 863
	Alkyl-Pi-alkyl	ALA 751, VAL 734, LYS 753, LEU 796
Cocrystal of 4EZ5	Pi-Sigma	PHE 1004, VAL 734, LEU 785
	Caron-hydrogen bond	ALA 751
	Alkyl-Pi-alkyl	LEU 726, LEU 852

Table S5: Biological activities of Curcumene

Pa	Pi	Activity
0.942	0.004	Mucomembranous protector
0.876	0.010	Ubiquinol-cytochrome-c reductase inhibitor
0.827	0.005	Phosphatidylcholine-retinol O-acyltransferase inhibitor
0.815	0.004	All-trans-retinyl-palmitate hydrolase inhibitor
0.781	0.005	Fatty-acyl-CoA synthase inhibitor
0.756	0.002	Plastoquinol-plastocyanin reductase inhibitor
0.754	0.008	Linoleate diol synthase inhibitor
0.739	0.004	Beta-carotene 15,15'-monooxygenase inhibitor
0.728	0.014	Fibrinolytic
0.722	0.048	CDP-glycerol glycerophosphotransferase inhibitor
0.717	0.007	Cholesterol antagonist
0.715	0.006	Adenomatous polyposis treatment
0.715	0.004	Gastrin inhibitor
0.700	0.016	Antiinflammatory

Table S6: Biological activities of Caryophyllene

Pa	Pi	Activity
0.915	0.005	Antineoplastic
0.897	0.005	Antieczematic
0.847	0.005	Apoptosis agonist
0.799	0.021	CYP2J substrate
0.792	0.003	Transcription factor NF kappa B stimulant
0.763	0.005	Antineoplastic (lung cancer)
0.760	0.004	MMP9 expression inhibitor
0.746	0.048	CYP2C12 substrate
0.745	0.011	Antiinflammatory
0.734	0.005	Antipsoriatic
0.734	0.006	Dermatologic
0.722	0.002	NF-E2-related factor 2 stimulant
0.709	0.011	Phosphatase inhibitor

Table S7: Biological activities of Bergamotene

Pa	Pi	Activity
0.847	0.011	CYP2J substrate
0.822	0.020	Testosterone 17beta-dehydrogenase (NADP+) inhibitor
0.774	0.005	Cardiovascular analeptic
0.766	0.016	Antineoplastic
0.739	0.012	Apoptosis agonist
0.721	0.004	Transcription factor NF kappa B stimulant
0.721	0.004	Transcription factor stimulant
0.700	0.006	Antineoplastic (lung cancer)

Table S8: Biological activities of Cyclohexene, 3-(1,5-dimethyl-4-hexenyl)-6-methylene-, [S-(R*,S*)]-

Pa	Pi	Activity
0.904	0.005	Antieczematic
0.827	0.009	Antineoplastic
0.805	0.017	Mucomembranous protector
0.789	0.019	Alkenylglycerophosphocholine hydrolase inhibitor
0.766	0.002	Retinol dehydrogenase inhibitor
0.760	0.012	Prostaglandin-E2 9-reductase inhibitor
0.750	0.004	Antipsoriatic
0.742	0.015	Protein-disulfide reductase (glutathione) inhibitor
0.720	0.004	Vitamin-K-epoxide reductase (warfarin-insensitive) inhibitor

Table S9: Biological activities of Benzene, 1-(1,5-dimethylhexyl)-4-methyl-,

Pa	Pi	Activity
0.894	0.006	Ubiquinol-cytochrome-c reductase inhibitor
0.891	0.008	Testosterone 17beta-dehydrogenase (NADP+) inhibitor
0.879	0.009	Phobic disorders treatment
0.870	0.009	Polyporopepsin inhibitor
0.861	0.010	Alkenylglycerophosphocholine hydrolase inhibitor
0.842	0.004	Anesthetic general
0.825	0.004	Cholestanetriol 26-monooxygenase inhibitor
0.829	0.015	Chymosin inhibitor
0.829	0.015	Saccharopepsin inhibitor
0.829	0.015	Acrocylindropepsin inhibitor
0.814	0.008	Alkylacetylglycerophosphatase inhibitor
0.774	0.004	Adenomatous polyposis treatment
0.767	0.004	Tpr proteinase (Porphyromonas gingivalis) inhibitor
0.772	0.014	Glutamyl endopeptidase II inhibitor
0.756	0.012	Prostaglandin-E2 9-reductase inhibitor
0.769	0.029	CYP2J substrate
0.743	0.003	Plastoquinol-plastocyanin reductase inhibitor
0.744	0.010	Fibrinolytic
0.743	0.025	Sugar-phosphatase inhibitor
0.724	0.010	Limulus clotting factor B inhibitor
0.708	0.008	Cholesterol antagonist
0.708	0.013	CYP2B6 substrate
0.696	0.006	Insulin promoter

Table S10: Biological activities of Tumerone

Pa	Pi	Activity
0.847	0.018	Ubiquinol-cytochrome-c reductase inhibitor
0.822	0.016	CYP2J substrate
0.766	0.010	Apoptosis agonist
0.776	0.025	Mucomembranous protector
0.759	0.015	HIF1A expression inhibitor
0.749	0.010	Phosphatidylcholine-retinol O-acyltransferase inhibitor
0.741	0.005	Carminative
0.730	0.008	All-trans-retinyl-palmitate hydrolase inhibitor
0.728	0.009	Fatty-acyl-CoA synthase inhibitor
0.751	0.039	Testosterone 17beta-dehydrogenase (NADP+) inhibitor
0.722	0.016	Fibrinolytic

0.698	0.028	Glutamyl endopeptidase II inhibitor
0.722	0.053	CYP2C12 substrate
0.678	0.009	Adenomatous polyposis treatment
0.656	0.010	MMP9 expression inhibitor
0.707	0.061	Aspulvinone dimethylallyltransferase inhibitor

Table S11: Biological activities of Curlone

Pa	Pi	Activity
0.859	0.009	Antieczematic
0.817	0.010	Antineoplastic
0.730	0.012	Apoptosis agonist
0.718	0.018	HIF1A expression inhibitor
0.695	0.017	Immunosuppressant
0.677	0.008	Carminative
0.717	0.049	Mucomembranous protector
0.711	0.050	Gluconate 2-dehydrogenase (acceptor) inhibitor
0.662	0.006	Antipsoriatic
0.658	0.013	Antifungal
0.621	0.002	Testosterone agonist

Table S12: Biological activities of (6R,7R)-Bisabolone

Pa	Pi	Activity
0.823	0.025	Ubiquinol-cytochrome-c reductase inhibitor
0.800	0.003	Beta-carotene 15,15'-monooxygenase inhibitor
0.812	0.018	CYP2J substrate
0.762	0.023	Alkenylglycerophosphocholine hydrolase inhibitor
0.737	0.002	Retinol dehydrogenase inhibitor
0.738	0.015	Alkylacetylglycerophosphatase inhibitor
0.738	0.015	Protein-disulfide reductase (glutathione) inhibitor
0.721	0.003	Plasmanylethanolamine desaturase inhibitor
0.721	0.004	CYP4A11 substrate
0.698	0.006	Prenyl-diphosphatase inhibitor
0.682	0.003	Antiviral (Rhinovirus)
0.701	0.022	Fibrinolytic
